# COVID-19-Associated Acute Limb Ischemia in a Patient on Therapeutic Anticoagulation

**DOI:** 10.7759/cureus.10655

**Published:** 2020-09-25

**Authors:** James C Gubitosa, Phoenix Xu, Ahmed Ahmed, Kathleen Pergament

**Affiliations:** 1 Internal Medicine, University Hospital - Rutgers New Jersey Medical School, Newark, USA

**Keywords:** therapeutic anticoagulation, covid 19, acute arterial thrombosis, disseminated intravascular coagulation (dic), sars-cov-2, fondaparinux, antithrombin iii, covid coagulopathy, hypercoagulable state, heparin-induced thrombocytopenia (hit)

## Abstract

Coronavirus disease 2019 (COVID-19), caused by severe acute respiratory syndrome coronavirus 2 (SARS-CoV-2), has been found to cause multiple complications across several organ systems in patterns not typically observed in previous iterations of the virus. Hemostatic mechanisms have been noted to be significantly altered in particular, resulting in a disseminated intravascular coagulation (DIC)-like picture with elements of coagulopathy as well as hypercoagulability.

A 65-year-old man with hypertension, hyperlipidemia, prior tobacco use, chronic kidney disease, and diabetes presented from a correctional facility with hypoxia. The diagnosis of COVID-19 was confirmed. With his elevated D-dimer of >7,955 ng/mL (reference: 90-500 ng/mL) in the setting of COVID-19 and hypoxia, he was empirically started on therapeutic anticoagulation with enoxaparin. His oxygen requirements increased, mental status deteriorated, and platelets began falling, raising concern for heparin-induced thrombocytopenia versus DIC. Heparin products were discontinued in favor of a direct oral anticoagulant. He later became obtunded and unable to tolerate oral medications. Fondaparinux was initiated. Two days later, he was found to have acute limb ischemia of the right lower extremity. He underwent surgical thrombectomy but required an above-the-knee amputation the following day. Shortly after he died secondary to hypoxic respiratory failure.

This case highlights the derangement of hemostatic mechanisms seen prominently in COVID-19 infection and raises questions as to appropriate anticoagulant choices to adequately prevent thrombosis. Thorough physical exams should be performed on all patients with COVID-19, taking into account this documented hypercoagulability. Further investigation is warranted into the use of heparin products as the anticoagulant of choice in these patients given observed deficiencies of antithrombin III (ATIII).

## Introduction

Coronavirus disease 2019 (COVID-19), caused by severe acute respiratory syndrome coronavirus 2 (SARS-CoV-2), has been found to cause multiple complications across several organ systems. Hemostatic mechanisms have been noted to be significantly deranged, resulting in a disseminated intravascular coagulation (DIC)-like picture with elements of coagulopathy as well as hypercoagulability present. In fact, the most common laboratory abnormalities seen in COVID-19-associated cases include an elevated prothrombin time and an elevated D-dimer [1-3]. Here, we report a case of a 65-year-old man who presented with acute hypoxic respiratory failure secondary to COVID-19. His hospital course was complicated by acute limb ischemia due to an acute occlusion of the right popliteal artery requiring eventual amputation of the limb, ultimately resulting in his death [4].

## Case presentation

**Figure 1:**
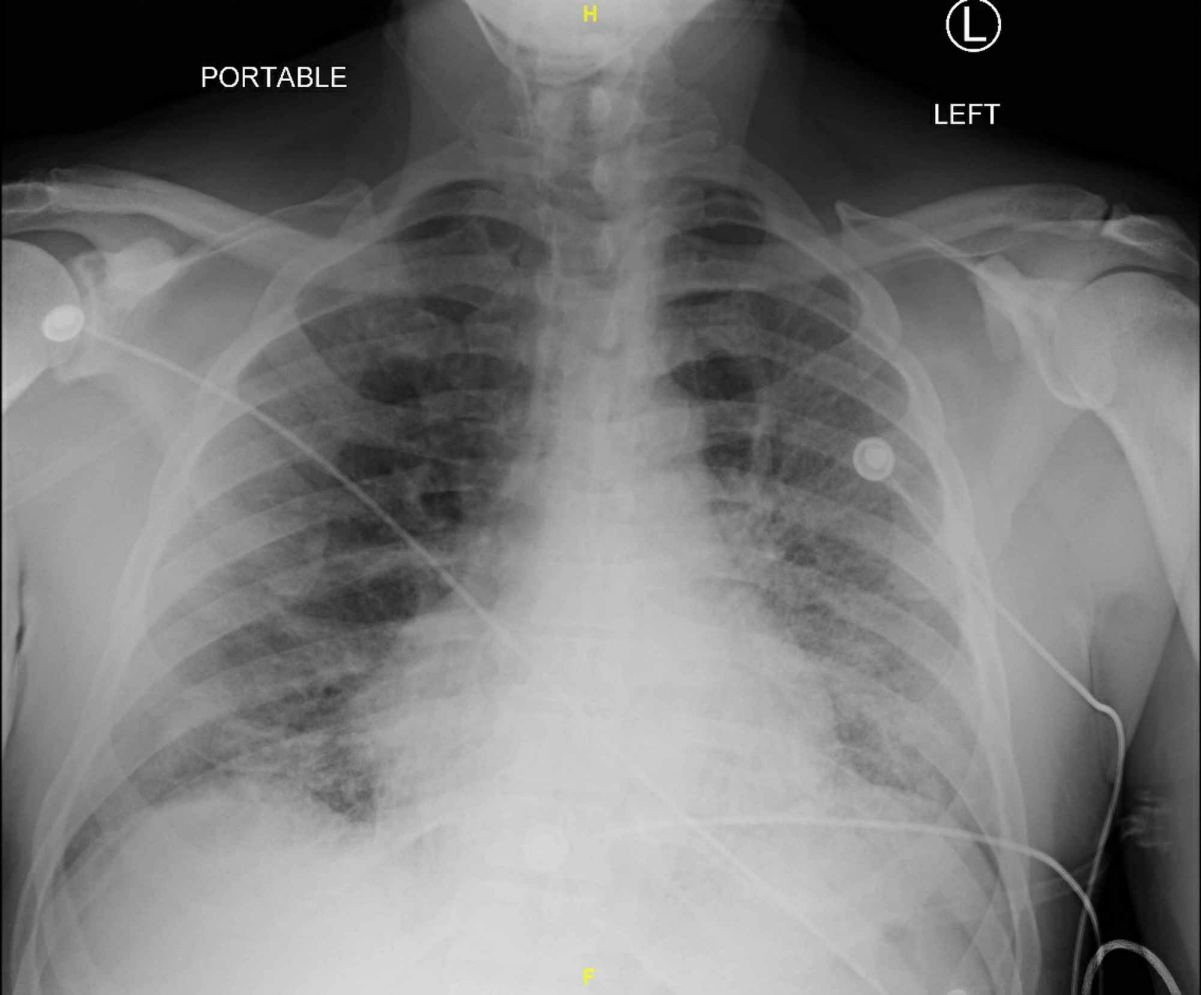
Posteroanterior (PA) chest X-ray upon admission Demonstrating bilateral pulmonary consolidations, more extensively on the left. Findings compatible with viral pneumonia.

**Figure 2:**
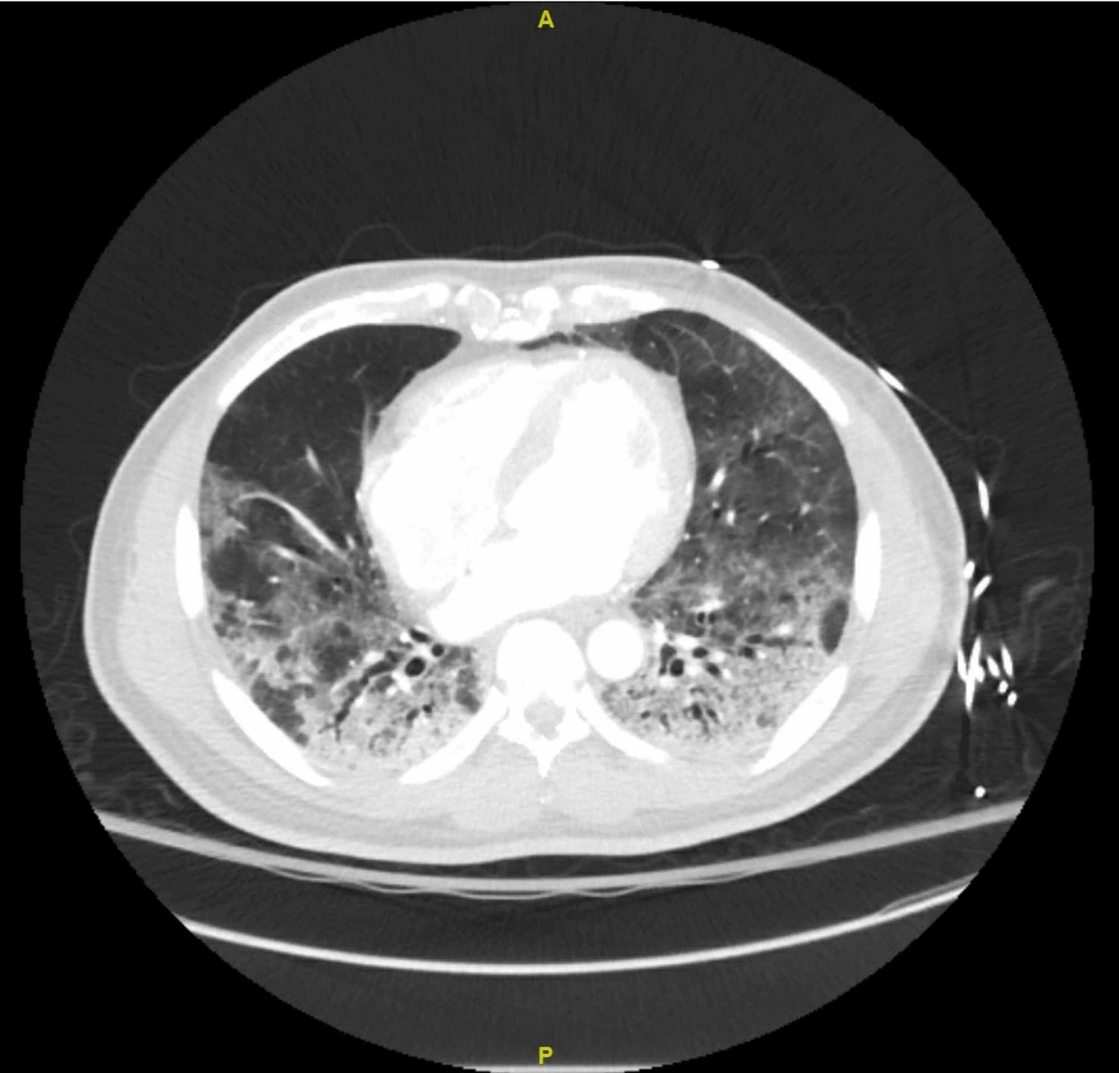
CT scan of the chest with intravenous (IV) contrast, transverse view Demonstrating diffuse ground-glass opacities in bilateral lung fields, most extensive in the bilateral lower lobes with mixed attenuation consolidations and accompanying traction bronchiectasis.

A 65-year-old man with a history of hypertension, hyperlipidemia, prior tobacco abuse, stage 2 chronic kidney disease (CKD), and diabetes presented from a correctional facility with progressive shortness of breath, cough, and fever for several days. These symptoms began insidiously and then rapidly worsened in the days prior to admission. Many inmates from the same prison were known to be hospitalized with COVID-19. In the emergency department, he was found to be hypoxemic, saturating at 83% on room air that improved with oxygen via non-rebreather mask. The patient’s chest X-ray demonstrated bilateral patchy opacities consistent with viral pneumonia (Figure 1). CT of the chest with intravenous (IV) contrast further characterized the bilateral ground-glass opacities (Figure 2). Labs were notable for elevated inflammatory markers lactate dehydrogenase (LDH) 1,153 U/L (reference: 120-250 U/L), ferritin 4,132 ng/mL (reference: 30-400 ng/mL), and D-dimer >7,955 ng/mL (reference: 90-500 ng/mL). Lymphopenia was also present on complete blood count. The diagnosis of COVID-19 was confirmed via nasal swab real-time polymerase chain reaction (RT-PCR).

Therapeutic anticoagulation was empirically initiated with subcutaneous enoxaparin (1 mg/kg twice daily) given the hypercoagulable state observed in many COVID-19 patients with elevated D-dimer. Enoxaparin was chosen based on the patient’s still adequate creatinine clearance (CrCl) of 73 mL/min and the ease of dosing compared to heparin. Three days after the first dose of enoxaparin, the patient’s platelet count began to fall at a moderate pace, dropping from 199 to 88 x 10^3^ platelets/µL (reference: 150-450 x 10^3^ platelets/µL) by hospital day 7. At the same time, the patient’s renal function began to worsen from a baseline creatinine of 1.4-3.0 mg/dL (reference: 0.7-1.2 mg/dL). Urinalysis demonstrated mild proteinuria with 100 mg/dL of protein detected (reference range: 0 mg/dL) as well as large blood (reference range: negative). Moreover, his fibrinogen levels fell below 100 mg/dL (reference: 145-490 mg/dL), and mental status continued to deteriorate. DIC was suspected given the patient’s clinical findings, but heparin-induced thrombocytopenia (HIT) could also not be ruled out. Qualitative anti-heparin antibodies were sent and found to be positive. Concern for HIT was still low given the precipitation of thrombocytopenia within four days instead of the classic 5, and a low 4T score of 2. Nonetheless, a serotonin-release assay (SRA) was sent but would take several days to return. Until the SRA had returned, heparin products were avoided, and therapeutic anticoagulation cautiously continued with oral apixaban. While superinfection and sepsis-induced coagulopathy were considered, serial blood and urine cultures remained negative. Over time, the patient’s mental status continued to worsen, and he became unable to safely tolerate oral medications. A CT of the head was ordered but was unable to be performed in a timely manner due to significant strain upon the facility’s scanners. A nasogastric tube was attempted but deemed unsafe as significant naso/oropharyngeal bleeding occurred upon manipulation. With no enteric access, therapeutic anticoagulation was instead switched to subcutaneous fondaparinux at a therapeutic renally calculated dose.

**Figure 3:**
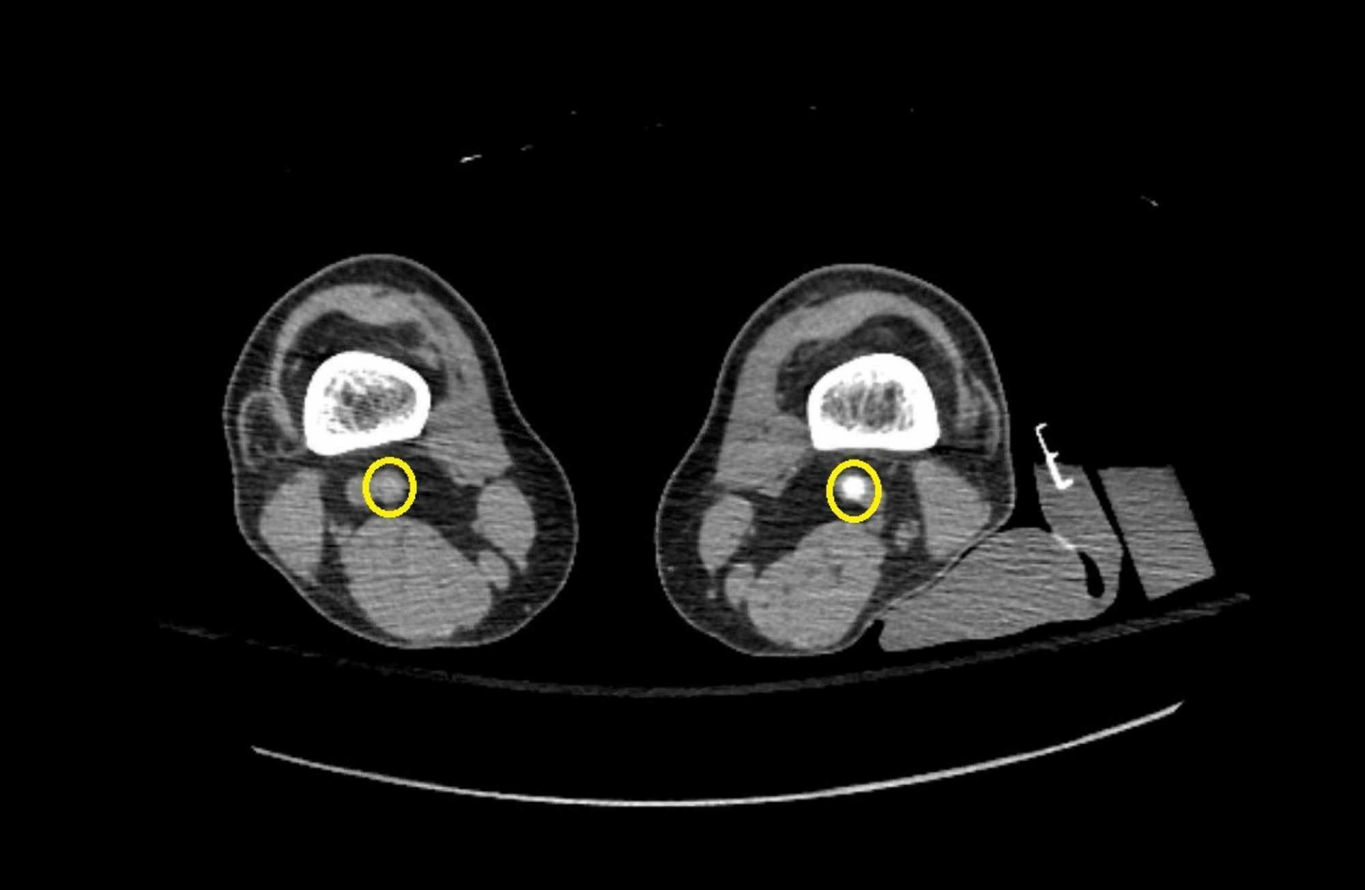
CT scan of the bilateral lower extremities with intravenous (IV) contrast Demonstrating acute occlusion of the right popliteal artery at the level of the distal femur/knee and patency of the left popliteal artery. Bilateral popliteal arteries are indicated by yellow circles.

The patient’s mental status and renal function began to improve over the following days. His respiratory status also improved, as he was being transitioned to a nasal cannula from a non-rebreather. The SRA also returned negative, effectively ruling out HIT. However, two days after initiation of the fondaparinux, the patient complained of acute right leg cramps and was noted to have a cold right leg on follow-up neurological examination. The leg appeared dark, dusky, and dry distal to the knee compared to the left leg. The patient was unable to appreciate light touch, pressure, pain, or temperature sensation distal to the right knee, and had limited ability to move his toes. Right dorsalis pedis pulses and posterior tibial pulses were absent on palpation and confirmed to be absent on evaluation with bedside doppler. Labs at the time were significant for a persistently elevated D-dimer of > 7,955 ng/mL (reference: 90-500 ng/mL), partial thromboplastin time (PTT) 30 seconds (reference: 24-34.2 seconds), international normalized ratio (INR) 2.0 (reference: 0.9-1.17), platelets 82 x 10^3^/µL (reference: 150-450 x 10^3^ platelets/µL), and fibrinogen 247 mg/dL (reference: 145-490 mg/dL). Vascular surgery was emergently consulted for suspected acute limb ischemia, and the patient was started on an argatroban infusion with plans for emergent operative intervention. A perioperative CT angiogram of the chest and lower extremities demonstrated no pulmonary embolism but complete acute occlusion of the right popliteal artery at the level of the knee (Figure 3). 

The patient was taken for operative thrombectomy with four-compartment fasciotomy of the right lower extremity. During the procedure, the patient was found to have viable musculature within the superficial and deep posterior compartments but non-viable muscle in all other compartments. Unfortunately, due to bleeding complications and worsening shock, the patient was taken for a right above-the-knee amputation the following day. Following the procedure, the patient was unable to be extubated due to increased oxygen requirements. He was admitted to the surgical intensive care unit for the management of his mechanical ventilation as well as worsening shock requiring pressor support. The patient experienced cardiac arrest shortly afterwards and passed away despite resuscitative measures. During his hospitalization, the patient received COVID-19 convalescent plasma twice, as well as tocilizumab. He was being considered for therapy with remdesivir just prior to his passing. 

## Discussion

This patient’s clinical course is remarkable in that he experienced an acute arterial thromboembolic event while on therapeutic (albeit appropriately renally dosed) anticoagulation. It is unclear how our patient developed an acute arterial ischemic event while on a therapeutic dose of anticoagulation. Although the patient was switched from multiple anticoagulants (enoxaparin to apixaban to fondaparinux), all doses were provided in a timely manner, with appropriate dosing, and without any gaps in anticoagulant coverage. In addition, the patient was not also taking any medications known to decrease the efficacy of fondaparinux and/or promote thrombosis.

COVID-19 has been found to affect almost every organ system and cause substantial morbidity and mortality. This patient experienced respiratory compromise as well as significant hematologic derangements resulting in a DIC-like picture with rapid consumption of coagulation factors as well as hypercoagulability. These factors precipitated significant morbidity and mortality with the loss of a limb, followed by his death. It is possible that the cause of his acute arterial thrombosis was DIC, but it is also possible that COVID-19 contributed to his hypercoagulability by a yet unknown mechanism. The prevalence of these arterial and venous thromboses in COVID-19 is well documented in the literature [1-3,5-8].

In a recent study of 184 patients hospitalized with confirmed COVID-19, 70% experienced coagulopathy, and 40% of those experienced some form of venous or arterial thromboembolic event [1]. A case series by Gonzalez et al. described three cases of peripheral arterial thrombosis in COVID-19 patients [7]. A retrospective study of venous thromboembolism (VTE) incidence in 26 patients with COVID-19 by Llitjos et al. demonstrated that 100% (n = 8) of those on prophylactic dose anticoagulation, and 56% (n = 10) of those on therapeutic dose anticoagulation experienced VTE [6]. A case report by Griffen et al. described an individual on therapeutic enoxaparin who also developed acute ischemia of the lower limb [8]. The risk of thromboembolic events and the sheer magnitude of COVID-19-associated admissions have prompted many hospitals to establish novel protocols with which these individuals are provided anticoagulation at increased or even therapeutic doses compared to the more common prophylactic dose. The exact mechanism through which COVID-19 promotes coagulopathy/hypercoagulability is still under investigation, as is the efficacy of these novel anticoagulation protocols.

Significant efforts have been made to identify the coagulopathic mechanism of COVID-19. Patients hospitalized with severe COVID-19 are subject to immobility that can commonly predispose to thrombosis. Acute phase reactants, including cytokines such as interleukin-6 (IL-6), are elevated and have been shown to cause endothelial damage and dysfunction, which can further predispose to thrombosis. Vascular findings in several patients with severe COVID-19 demonstrated endothelitis, with viral inclusion bodies seen under light microscopy in the endothelium of the specimens [9]. Skin and lung biopsies from five patients were examined in a separate study and found to have deposition of C5b-9, C4d, and MASP2 in their associated microvasculature with resultant microvascular damage. These findings were consistent with profound and generalized activation of both alternative and lectin-based complement pathways [10].

One study of 94 patients with confirmed COVID-19 demonstrated a statistically significant relative deficiency of antithrombin III (ATIII) compared to control (p < 0.001) [11]. ATIII targets and inactivates proteases of the clotting cascade, namely factors X, IX, XI, XII, and most importantly factor II, also known as thrombin. This inactivation leads to decreased coagulation. The anticoagulants heparin, enoxaparin, and the less commonly used fondaparinux potentiate ATIII as their primary measure of promoting anticoagulation. Individuals with hereditary or acquired ATIII deficiency (as in the case of COVID-19 patients) may thereby experience decreased efficacy of these anticoagulants.

A more recent study analyzed whole blood samples of 24 COVID-19 patients using thromboelastography (TEG). TEG demonstrated decreased clotting time from the point of coagulation ignition to the appearance of the clot (decreased R-value), increased velocity of clot formation (decreased K-value and increased K-angle), as well as increased values of clot maximum amplitude (MA). Again, decreased ATIII levels, as well as increased fibrinogen, D-dimer, factor VIII, and von Willebrand factor were observed. These values were all consistent with a hypercoagulable state [12].

Nephrotic syndrome is also known to cause an acquired ATIII deficiency via renal protein loss, defined as 3-3.5 g of urine protein in 24 hours [13]. Our patient not only had pre-existing CKD, but also demonstrated mild proteinuria during his hospitalization, with a spot-urine protein of 100 mg/dL (reference: 0 mg/dL). Had the patient not passed away, he may have benefitted from a 24-hour urine protein collection to diagnose nephrotic syndrome. No prior records were available to further characterize the severity and nature of his CKD. It is plausible that an ATIII deficiency was precipitated by COVID-19-induced kidney injury/subsequent nephrotic syndrome, but this phenomenon has yet to be thoroughly investigated. 

It is also important to note that our patient had numerous risk factors for vascular disease, including hyperlipidemia, hypertension, diabetes, prior tobacco abuse, and his CKD. Aortoiliac atherosclerotic disease was noted on the perioperative CT angiogram as well. It was known that his comorbidities were poorly controlled. Outpatient blood pressure readings were approximately 160/90 mmHg on average, his hemoglobin A1c was 12.6% (reference: 4.8%-5.9%), his low-density lipoprotein (LDL) was 162 mg/dL (reference: 0-99 mg/dL), and his calculated 10-year atherosclerotic cardiovascular disease (ASCVD) risk was 46.0%. He had long struggled with blood pressure and glycemic control, and it is likely these contributed to his development of CKD. Overall, his history paints the picture of a very unhealthy vascular state, one that may have predisposed to inflammation and thrombosis contributing to his mortality.

At the time of composing this article, there are no established guidelines for preventing/managing “COVID-19-associated coagulopathy” or “CAC” as it is sometimes called. A study of 449 COVID-19 patients demonstrated a statistically significant decrease in mortality (40.0% experimental, 64.2% control) in those receiving prophylactic doses of heparin and enoxaparin [14]. However, given the persistently high mortality rate and incidence of thrombosis even while on prophylactic dose anticoagulation, organizations and institutions have developed novel protocols to determine if select patients should instead receive full-dose or therapeutic anticoagulation [15]. The International Society for Thrombosis and Hemostasis (ISTH) has developed an interim set of guidelines that recommends for as-usual prophylactic anticoagulation in hospitalized patients but consideration for therapeutic anticoagulation in patients who are deemed critically ill [16]. Many hospitals have taken further measures to gauge the risk of thrombosis, with most based off the D-dimer level. At these institutions, a significantly elevated D-dimer (at least >2,000 mg/dL) calls for an automatic upgrade to therapeutic anticoagulation. This thought process is what lead to our patient being started on therapeutic anticoagulation.

Multiple studies are under way to thoroughly investigate the various sequelae of infection with COVID-19. For CAC, research in molecular microbiology and biochemistry will be pivotal. With this information, we can more fully understand the molecular mechanics of how COVID-19 causes this hypercoagulable state and intervene with the appropriate choice of anticoagulation to counterbalance the hematologic disruption caused by the virus.

## Conclusions

Arterial thrombosis in coagulopathic states such as DIC and CAC has been cited in the literature; however, it is uncommon. Arterial thrombosis occurring while a patient is on anticoagulation is even less common. In this patient, the presence of COVID-19 and its associated derangement of hemostatic mechanisms cannot be ignored as a possible exacerbating factor of his thrombosis. There is a clear association between COVID-19 and thrombotic events. Moreover, with presentations of COVID-19 being so novel, this case demonstrates the possibility that certain forms of anticoagulation, such as fondaparinux, could be inferior in preventing a thrombotic event. Given the cited relative deficiency of ATIII in CAC and nephrotic syndrome, one can hypothesize that an acute arterial thrombosis may have been avoided if this patient was started on an argatroban infusion from the beginning of his hospital course, which would have provided therapeutic anticoagulation independent of ATIII levels. Further research is needed to assess the efficacy of and indications for the different anticoagulants in the setting of hypercoagulable states and elevated risk for thrombosis in the COVID-19 infection. Clinicians caring for COVID-19 patients should be aware of this hypercoagulable state and take necessary measures to prevent and treat its occurrence.
